# Benchmarking of aggregated length of stay after open and laparoscopic surgery for cancers of the digestive system

**DOI:** 10.1002/bjs5.67

**Published:** 2018-04-23

**Authors:** K. Lassen, L. S. Nymo, F. Olsen, K. Søreide

**Affiliations:** ^1^ Department of Hepato‐Pancreato‐Biliary Surgery Oslo University Hospital Oslo Norway; ^2^ Department of Gastrointestinal Surgery University Hospital of North Norway Tromsø Norway; ^3^ Institute of Clinical Medicine University of Tromsø Tromsø Norway; ^4^ Centre for Clinical Documentation and Evaluation Northern Norway Regional Health Authority Tromsø Norway; ^5^ Department of Clinical Medicine University of Bergen Bergen Norway; ^6^ Department of Gastrointestinal Surgery Stavanger University Hospital Stavanger Norway; ^7^ Department of Clinical Surgery Royal Infirmary of Edinburgh, and University of Edinburgh Edinburgh UK

## Abstract

**Background:**

Length of hospital stay (LOS) may serve as a surrogate measure of healthcare quality and resource use, particularly when transfers of care and readmissions are accounted for. This study aimed to benchmark true hospital stay by measuring index, transfer and readmission stays across the range of digestive cancer surgery.

**Methods:**

A cohort study of all patients undergoing resection for cancer of the oesophagus, stomach, liver, pancreas, colon or rectum in 2012–2016 was undertaken. Index LOS, transfer and readmission stays were merged into an ‘aggregated’ length of stay (a‐LOS), and compared between organ sites and between open and minimal‐access approaches.

**Results:**

In total, 24 354 resections were reported (mean age of patients 68·3 years; 51·3 per cent were men). Resections were reported as laparoscopic for 9151 procedures (37·6 per cent), with a further 283 (3·0 per cent) described as converted to open surgery. Use of a‐LOS compared with standard LOS added a median of 5 days for pancreatoduodenectomy, 4 days for major liver resections, 3 days for oesophageal and gastric resections, and 2 days for minor liver, distal pancreatic and rectal resections.

**Conclusion:**

Overall hospital stay across organ sites and procedures is better described by a‐LOS. The study benchmarks the use of total hospital days during the first 30 days in a universal healthcare system.

## Introduction

The annual number of resections for cancers of the digestive tract has a profound impact on the use of healthcare resources. Several measures have been used to examine the use of resources in an effort to develop quality improvement initiatives. Functional recovery after surgery is important for patients and carers^1^ ^2^, although it may be difficult to assess objectively. Postoperative length of hospital stay (LOS) is a readily available surrogate endpoint, often assumed to be a measure of recovery, although open to criticism^3^. The focus on financial savings with the emergence of laparoscopic approaches to gastrointestinal surgery has nevertheless consolidated a focus on LOS as a meaningful outcome, especially when it is shorter than anticipated^4^ ^5^. Importantly, transfers of care within or between healthcare facilities and readmissions should be accounted for, especially when the planned duration of the index stay is short. Available data, however, tend to be derived from trials and academic centre series, and fail to consider resource issues that influence LOS in the average hospital, or exclude elderly patients and those with major co‐morbidities^4^, ^5^, ^6^, ^7^, ^8^.

The aim of this study was to create a benchmark of total hospital stay by proposing an amalgamated metric of index stay, stays related to transfers of care and readmissions into an ‘aggregated’ LOS across the range of gastrointestinal cancer surgery. By including a complete 5‐year national cohort, such a measure would offer insight into the relative use of healthcare services by each resection group, between cancer sites and for surgical access type. This might then serve as a real‐life reference for future clinical series and trials focusing on LOS as an outcome measure.

## Methods

The investigation was carried out and reported in accordance with the STROBE guidelines^9^.

The Centre for Clinical Documentation and Evaluation holds a concession from the Norwegian Data Protection Authority to access data from the Norwegian Patient Register (NPR) for patients treated at Norwegian hospitals in the period 1 January 2012 to 31 December 2016. Encrypted patient serial numbers make it possible to describe patient pathways involving all hospitals over several years.

All patients with a procedure code within the NPR denoting surgical resection for cancer of the oesophagus, stomach, liver, pancreas, colon or rectum in the period from 2012 to 2016 were included. For the index year 2015^10^, the number of hospitals that did any specific number of cancer operations for the mentioned organ sites was identified, as an estimate of centralization and volume provision across the country.

### The healthcare programme and the database

Norway has a universal healthcare programme for all citizens (population almost 5·3 million) that ensures equal access to care. Some gastrointestinal cancer surgery is centralized to a limited number of centres, and postoperative transfer to local hospitals is used frequently. Every citizen has a unique 11‐digit social security number that can be tracked between several registries and healthcare records, given appropriate permissions. Surgery for cancer of the digestive system in Norway is performed exclusively at public healthcare hospitals, and all Norwegian hospitals must submit data to the NPR for registration and reimbursement purposes. The selected NPR variables have good data quality and completeness^11^, and also illustrate core quality outcome metrics in major surgery. The relatively large sums reimbursed for major surgery ensure optimal data quality compared with, for instance, short admissions for investigation with no interventions.

A database of surgical procedures to include postoperative LOS related to an index procedure, stays related to transfers and readmissions was created based on data extracted from the NPR. This registry enables individual patients to be tracked from one stay to another, allowing for identification of a subsequent readmission or following planned transfer from the hospital where index surgery was performed.

### Inclusion criteria

All resections of the oesophagus, stomach, liver, pancreas, colon and rectum in the 5‐year interval from 1 January 2012 to 31 December 2016 were included. Operations were identified from the Nordic Medico‐Statistical Committee (NOMESCO) Classification of Surgical Procedures (NCSP), version 2014^12^. Liver resections were further subdivided into major or minor resections according to Brisbane 2000 terminology^13^, where major resection indicated excision of three consecutive segments or more.

It was assumed that virtually all resections of the oesophagus, stomach, liver, pancreas and rectum would have been performed for malignant or premalignant conditions. For colonic resections, only those for which a corresponding diagnosis of colonic cancer had been made within 30 days before or 30 days after resection were included. Resections performed within 30 days after other eligible resections were excluded, as these were considered reoperations. A ranking system was used when patients underwent resection of more than one organ. The ranking list was: oesophageal, pancreatoduodenal, rectal, gastric, distal pancreatic, colonic and liver resections. The higher‐ranked resection was included and the lower‐ranked resection ignored when both were recorded on the same date.

### Descriptors

The defined process variables were organ site of resection (oesophageal, gastric, liver, pancreatic, colonic or rectal) and access modality (open and laparoscopic resections had unique codes), as described over time for the study period. The number of hospitals providing the services was recorded.

### Reported outcomes and definitions

LOS was counted as the number of nights in hospital. All stays in the database (containing an eligible index operation) were coupled via the unique patient identifier with any subsequent stay at any Norwegian hospital with an admission date within 30 days of index operation.

The outcomes chosen were: conventional LOS at the institution performing the index procedure (including transfers of care within that institution); LOS after transfer to any other hospital; and LOS related to readmissions to either the index or any other hospital. Summation of these stays created an aggregated length of hospital stay (a‐LOS). Along with the median value, a‐LOS was further characterized with interquartile denominators (25th and 75th percentiles).

Conventional LOS was defined as the time from the index procedure to first discharge or transfer. a‐LOS was defined as the sum of all nights in any hospital within 30 days of the date of the index operation, under the assumption that planned stays for other conditions in the period immediately after major digestive tract surgery would be virtually non‐existent. A transfer stay was defined as admission to another hospital on the same date as discharge from the hospital performing the index procedure. A readmission was defined as admission to any hospital within 30 days of the index surgery, separated by an interval of at least one night from any index or transfer stay.

Surgical access was assigned as open unless a designated code for a laparoscopic resection had been used. Where patients had both a laparoscopic and an open resection code at index surgery, the operation was grouped as ‘converted’ (laparoscopic to open).

### Statistical analysis

Descriptive statistics were used as these represent a complete cohort of national data. Data are reported as median (i.q.r.) values.

## Results

**Figure 1:**
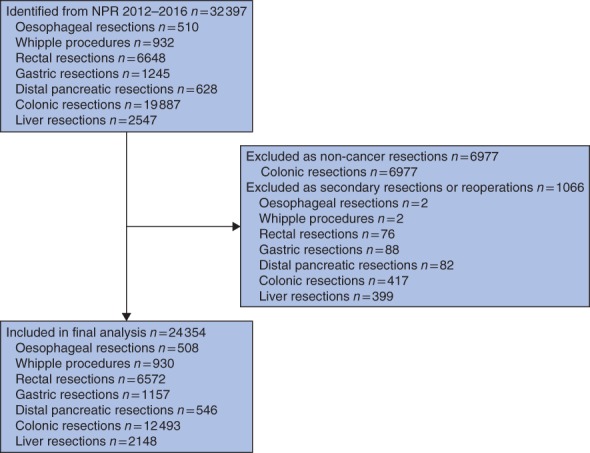
Flow chart of data extraction results. NPR, Norwegian Patient Register

Over the 5‐year interval, 32 397 oesophagogastric, hepatopancreatobiliary or colorectal resections were performed in Norway. Of these, 6977 colonic resections that were not coupled with a diagnostic code of colonic malignancy were excluded, along with a further 1066 procedures performed as secondary resections during another (higher ranked) resection or as reoperations, leaving a cohort of 24 354 resections for analysis (*Fig*. 1). Of these, 12 499 (51·3 per cent) of the patients were men, and the mean age overall was 68·3 years.

In the index year of 2015, 32 hospitals in Norway were doing colonic resections on a regular basis (more than 10 patients per year); 28 of these did more than 20 colonic resections for cancer. Other resections involved fewer hospitals, leaving 20 hospitals regularly performing rectal resections (more than 10 patients in 2015). Seven hospitals did gastric resections, with mean yearly case volumes ranging from 12 to 45, and five did liver and pancreatic surgery. For distal pancreatic resections the volumes per year ranged from 4 to 40, and for Whipple procedures from 11 to 103. The mean number of liver resections per hospital per year in the period ranged from 20 to 218^11^. Four hospitals did oesophageal resections, with the mean number per hospital per year ranging between 13 and 48^11^.

### Rates of laparoscopic and open procedures

Resections were laparoscopic in 9151 (37·6 per cent) of the procedures, with an additional 283 (3·0 per cent) of the total 9434 laparoscopic procedures converted to open surgery (Table 1). Overall, the proportion of patients operated on by laparoscopic approaches increased from 28·2 to 46·7 per cent in the study period. For the entire 5‐year interval, a laparoscopic resection code (with no combination with open‐access code) was reported in 235 (46·3 per cent) of the oesophageal resections, 246 (21·3 per cent) of the gastric resections, 5494 (44·0 per cent) of the colonic resections, 2294 (34·9 per cent) of the rectal resections, 559 (26·0 per cent) of the liver resections and 323 (59·2 per cent) of the distal pancreatic resections (Table 1). All 930 pancreatoduodenectomies were performed by open access.

**Table 1:** Components of aggregated length of hospital stay

	No. of patients	Median index LOS*, †	Transfer stay		Readmission stay		Median (i.q.r.) a‐LOS*
			% transferred	Median LOS*	% readmitted	Median LOS*	
Oesophageal resections							
Laparoscopic	235	15	28·5	7	8·9	1	18 (14–24)
Open	271	15	37·3	6	10·3	3	20 (14–30)
Converted	2	19·5	50	5	0	–	22 (14–30)
Total	508	15	33·3	6	9·6	2	18 (14–30)
Whipple procedures							
Open	930	9	56·8	5	12·4	4	14 (10–21)
Total	930	9	56·8	5	12·4	4	14 (10–21)
Rectal resections							
Laparoscopic	2294	6	11·4	5	12·6	4	6 (4–11)
Open	4211	8	15·4	6	14·6	3	10 (7–16)
Converted	67	7	24	6	16	2	10 (7–14)
Total	6572	7	14·1	6	13·9	3·5	9 (6–14)
Gastric resections							
Laparoscopic	246	6	22·0	4	9·3	7	8 (5–13)
Open	904	9	32·2	6	11·8	4	12 (8–19·5)
Converted	7	7	43	14	29	9	13 (6–27)
Total	1157	8	30·1	6	11·4	4	11 (7–18)
Distal pancreatic resections							
Laparoscopic	323	5	24·1	4	20·1	5	7 (5–11)
Open	215	9	25·1	6	18·6	4·5	13 (8–19)
Converted	8	6	38	4	25	4	9 (7–9·5)
Total	546	7	24·7	5	19·6	5	9 (5–14)
Colonic resections							
Laparoscopic	5494	4	4·7	5	9·3	3	5 (3–7)
Open	6820	7	9·5	6	11·4	4	8 (6–13)
Converted	179	6	9·5	4	11·7	3	7 (5–11)
Total	12 493	6	7·4	6	10·5	4	6 (4–10)
Major liver resections							
Laparoscopic	37	5	24	4	27	2	5 (3–16)
Open	564	8	51·1	5	14·5	4	12 (8–19·5)
Converted	6	5	50	6	17	7	7·5 (5–12)
Total	607	8	49·4	5	15·3	3	12 (8–19)
Minor liver resections							
Laparoscopic	522	2	15·7	3	12·8	4	3 (2–5)
Open	1005	6	32·8	4	13·4	3	8 (5–12)
Converted	14	4	29	3·5	0	–	5 (3–8)
Total	1541	4	27·0	4	13·1	4	6 (3–10)
Total liver resections							
Laparoscopic	559	2	16·3	3	13·8	4	3 (2–5)
Open	1569	7	39·4	4	13·8	3	9 (6–14)
Converted	20	5	35	4	5	7	5 (3·5–10)
Total	2148	5	33·3	4	13·7	4	7 (4–13)
Total resections							
Laparoscopic	9151	5	8·9	4	10·8	4	5 (3–9)
Open	14 920	8	19·3	6	12·7	4	9 (6–15)
Converted	283	6	16·6	5	13·1	2	8 (6–12)
Total	24 354	7	15·4	5	12·0	4	8 (5–13)

* Stays are measured as number of nights in hospital;

† index stay is from date of surgery. LOS, length of hospital stay; i.q.r., interquartile range; a‐LOS, ‘aggregated’ length of stay.

### Length of stay, transfers and readmissions

Conventional length of stay (postoperative index stay) is presented in Table 1 along with rate and, as applicable, median duration of stays related to transfers of care and readmission.

**Figure 2:**
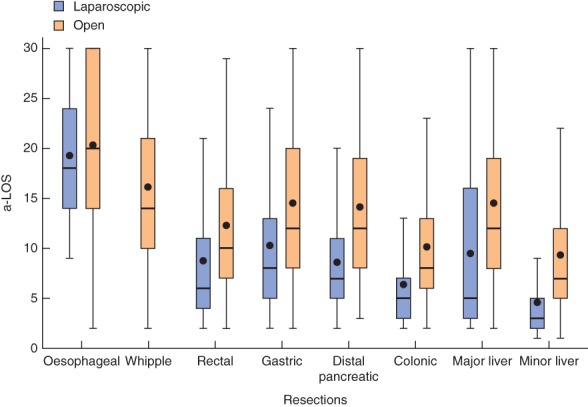
Box and whisker plot of aggregated length of hospital stay (a‐LOS) for eight resection groups. Bars within boxes show median values (quartile (Q) 2); boxes show interquartile ranges (Q3–Q1); black dots indicate mean values; upper whiskers denote Q3 + (i.q.r. × 1·5); lower whiskers denote the lowermost value within Q1 − (i.q.r. × 1·5). Stays are truncated at 30 days and hence means are skewed. For simplicity, converted cases are included in the open‐access group

**Figure 3:**
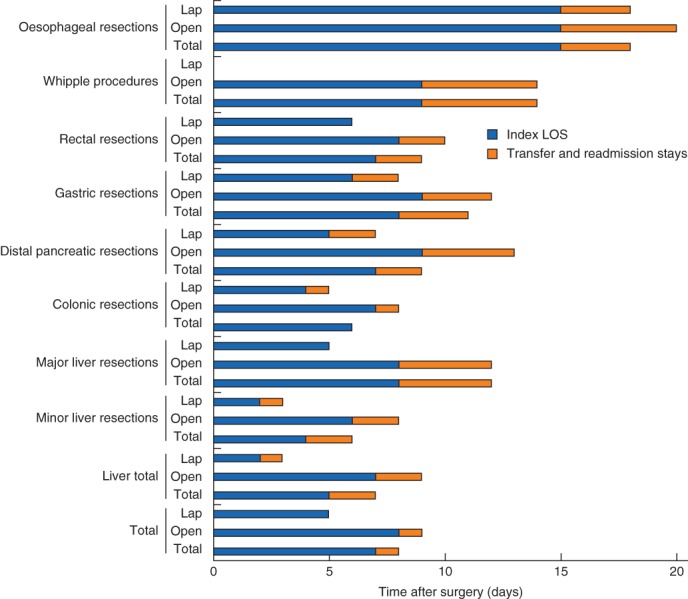
Relative components of aggregated length of hospital stay (a‐LOS): index LOS and added stays from transfers and readmissions combined. For simplicity, converted cases are not shown. All values are median. Lap, laparoscopic

The total length of all stays (a‐LOS) combining index, transfers and readmissions for the various organ resections is presented in Table 1 and Figs 2 and 3. The relative difference in LOS between laparoscopic and open groups increased substantially when a‐LOS was used instead of standard LOS for all resection groups except colonic surgery. The highest crude number of days in hospital added by the use of a‐LOS was 5 days for Whipple resections (median a‐LOS 14 days versus 9 days for median index LOS). Although crude numbers were lower, the relative increase in hospital days from conventional LOS to a‐LOS was 50 per cent for both open major liver resections and laparoscopic minor liver resections.

The rate of transfer stays was highest for the most centralized procedures and lowest for colonic resections. Readmission rate was highest for distal pancreatic resections at 19·6 per cent and lowest for oesophageal resections (9·6 per cent). The length of index stay after open distal pancreatic resections was almost twice that after laparoscopic access (9 *versus* 5 days respectively), but the transfer stay and readmission rates were almost identical at about 25 and 19 per cent respectively (*Table* 1).

## Discussion

This study provides an overview of the total use of hospital beds during the first 30 days after surgery for gastrointestinal cancer in Norway. Importantly, this is a complete national cohort and reflects unselected outcome of major cancer surgery performed in modern enhanced‐recovery settings in a universal healthcare system. Except for pancreatoduodenectomy, laparoscopic access increased across the range of gastrointestinal cancer surgery and accounted for nearly half of all procedures at the end of the study period.

The cohort included many patients often excluded from clinical trials, such as the elderly, frail, and those with co‐morbidities and obesity. Thus it offers a backdrop against which to compare control groups in clinical trials and provides a means to validate the generalizability of trial results. Detailed information on co‐morbidity was not available in this administrative data set, and adjusting for case mix was not possible.

For all resection groups in this study, laparoscopic access was associated with shorter index and aggregated LOS. This does not provide evidence that laparoscopy results in quicker recovery. Non‐randomized observational data cannot exclude the possibility of bias from imbalance in selection or clinical prejudices that these patients can be discharged sooner. The data do show, however, that patients who are treated with laparoscopic cancer surgery in Norway have shorter stays than those treated with open access.

LOS is a widely reported outcome measure in the era of laparoscopy, but subject to the criticism that it reflects not only functional recovery but also organizational structure and logistics^3^. In addition, short LOS has been associated with increased rates of readmission^14^. In the present series, with between‐procedures comparisons, shorter stays were not related to an increased readmission rate. For example, colonic resections had the shortest a‐LOS, but also a low rate of readmissions as a group. The present data show that, except for major liver and distal pancreatic resections, laparoscopic access also was associated with a lower rate of readmission. Patterns related to stay and readmission probably reflect procedure‐specific complications, management pathways and disease processes related to each type of surgery.

The strength of the present data is the ability of the NPR‐encrypted identity strings to capture all stays and readmissions anywhere in the country. Adding up every night spent in hospital within 30 days from index surgery to an a‐LOS provides a more accurate picture of resource use and extent of recovery.

An obvious finding in the present study was that across the range of gastrointestinal cancer surgery a‐LOS was much longer than LOS reported in RCTs or single‐centre series^4^ ^5^, ^15^. Seminal series have showed that open colectomy stays can be reduced to 2 days^4^, and laparoscopic surgery to just 23 h^5^. Although these may have been intended as ‘proof of principle’, the present data showed a median a‐LOS of 8 days for open and 5 days for laparoscopic colonic resections. A recently published RCT^15^ comparing laparoscopic with open minor liver resections reported a median stay of 2 days after laparoscopy and 4 days following open surgery. The present study, with a median a‐LOS of 8 days after open and 3 days after laparoscopic resection for minor liver resections, is an interesting contrast, suggesting selection bias in a real‐world setting, but equally providing the impetus to improve perioperative care, particularly for those undergoing open surgery.

Increasing LOS as a result of transfers of care clearly reflects degrees of centralization and planned LOS. It is not obvious whether this is a negative outcome from the patients' viewpoint. Only transfers and readmissions leading to at least one night in hospital were recorded in the present study. The large difference in the extent of transfer between the different types of resection reflects centralization. More than half of the patients undergoing pancreatoduodenectomy had transfer stays, reflecting the Whipple procedure being done in only five hospitals with high reliance on secondary care hospitals in the recovery phase. The rate of transfer stay was much lower for colonic surgery, where most patients received all care to discharge at the primary treatment site. These differences should be kept in mind when comparing trial results and outcomes reported from large tertiary referral centres, as the likelihood of prolonged care outside the primary institution may be high, yet remains unreported.

The present data set did not provide any information about the perioperative routines employed in the various hospitals. Although compliance data with enhanced recovery after surgery (ERAS) protocols were not available, most core items are considered to be well implemented in Norway, reflecting communications at national symposia, involvement in the ERAS^®^ Society guidelines and consensus papers^16^, ^17^, ^18^, ^19^, ^20^.

Some limitations to the study should be noted. The NPR was designed primarily for reimbursement purposes, and only selected items related to outcome are obtainable. At the beginning of the study period, a proportion of laparoscopic procedures may have been coded as open, as the coding system had a delay in revisions. Some surgeons may not have identified a proper laparoscopic code in the manual and used an open‐access code. The data for conversion from laparoscopic to open access are based on the presence of both laparoscopic and open‐access procedure codes at index surgery. This might have resulted in an underestimation of conversion rates if not followed. For some procedures, the overall numbers were small (only 37 laparoscopic major liver resections), and trends in access‐related outcome should be viewed carefully. For oesophageal resections, the access to two compartments further complicated data extraction. In the majority of ‘laparoscopic/thoracoscopic’ procedures, access to at least one compartment involved open surgery.

Despite these limitations, a‐LOS has been shown to be a more reliable marker of resource use after gastrointestinal cancer surgery. Measuring LOS related simply to the index procedure is inadequate for many of these operations.
